# Acceleration in BMI gain following COVID‐19 restrictions. A longitudinal study with 7‐ to 10‐year‐old primary school children

**DOI:** 10.1111/ijpo.12890

**Published:** 2022-01-26

**Authors:** Gerald Jarnig, Johannes Jaunig, Reinhold Kerbl, Volker Strenger, Gabriele Haeusler, Mireille N. M. van Poppel

**Affiliations:** ^1^ Institute of Human Movement Science, Sport and Health University of Graz Graz Austria; ^2^ Department of Pediatrics and Adolescent Medicine LKH Hochsteiermark/Leoben Austria; ^3^ Department of Paediatrics and Adolescent Medicine, Division of Paediatric Pulmonology and Allergology Medical University of Graz Graz Austria; ^4^ Department of Paediatrics and Adolescent Medicine, Division of General Paediatrics Medical University of Graz Graz Austria; ^5^ Department of Pediatrics and Adolescent Medicine, Division of Pulmonology, Allergology and Endocrinology‐ Vienna Bone and Growth Center Medical University of Vienna Austria

**Keywords:** BMI, children, COVID, obesity, overweight, primary school

## Abstract

**Background:**

The ramifications of COVID‐19 restrictions might accelerate the already rising proportion of children with overweight or obesity.

**Objectives:**

To assess the association between COVID‐19 restrictions and changes in body mass index (BMI) and the proportion of children with overweight or obesity.

**Methods:**

Cohort study with baseline measurements in September 2019 (prior to COVID‐19 restrictions) and follow‐up in June 2020, September 2020, and March 2021 at 12 primary schools in Austria. The height and weight of 738 children aged 7 to 10 years were measured and age‐ and sex‐specific national and international standardized values were calculated. Changes over time were analysed by analysis of variance.

**Results:**

Mean BMI_IOTF_ standard deviation scores (SDS) increased by 0.24 (95% CI, 0.21–0.28) between September 2019 and March 2021. The proportion of children with overweight or obesity increased from 20.7% to 26.2% during this period (*p* < 0.001) using national reference values—EQUI BMI_AUT_—comparable results were observed. Simultaneously, the height_AUT_ SDS increased by 0.06 (95% CI, 0.05–0.08) with a larger increase in girls (+0.11; *p* < 0.001) than in boys (+0.03; *p* = 0.19).

**Conclusions:**

COVID‐19 restrictions were associated with accelerated increases in mean BMI and the proportion of children with overweight or obesity. The increase in height SDS in girls calls for further investigations.

## 1 INTRODUCTION

When the COVID‐19 pandemic started to spread across the globe in Spring 2020,^1^ it combined with the pre‐existing pandemics of noncommunicable diseases which are related to physical inactivity and the increase in the proportion of children with overweight or obesity.^2^, ^3^, ^4^

The risk of COVID‐19 to overburden the healthcare system led governments around the world to impose restrictions to reduce COVID‐19 spread.^5^, ^6^ These restrictions resulted also in severely reduced options for physical activity for all age groups,^5^ and numerous studies reported subsequent changes in leisure‐time physical activity in children during the COVID‐19 pandemic.^7^, ^8^, ^9^ Low levels of physical activity are closely associated with an increased risk for children to be affected by overweight or obesity.^10^, ^11^, ^12^, ^13^ There are initial findings, that leisure‐time behaviour and physical activity during COVID‐19 restrictions were different in urban and rural areas.^14^

We previously reported a dramatic development of objectively measured body mass index (BMI) and cardiorespiratory fitness after the first lockdown in Spring 2020.^15^ Here, we follow the further development of BMI of primary school children in the area of Klagenfurt, Austria, between September 2019 and March 2021 and add data on height development. In addition, differences between children from urban and rural schools are assessed.

## 2 METHODS

The original aim of this study was to conduct a randomized controlled trial in primary school to describe the effects of a 1‐year intervention on the fitness and health status of children. In October 2019, the intervention was started and in the intervention group, all sports lessons in school were carried out by external coaches. The number of sports lessons remained the same as in the control group. In early 2020, the intervention had to be discontinued due to the COVID‐19 pandemic. Anthropometric data were nevertheless recorded and are now analysed as those of a longitudinal observation. The study was approved by the Research Ethics Committee of the University of Graz, Styria, Austria (GZ. 39/23/63 ex 2018/19) and registered in the German Clinical Study Register (ID DRKS00023824).

### 2.1 Selection of schools and participants

**FIGURE 1:**
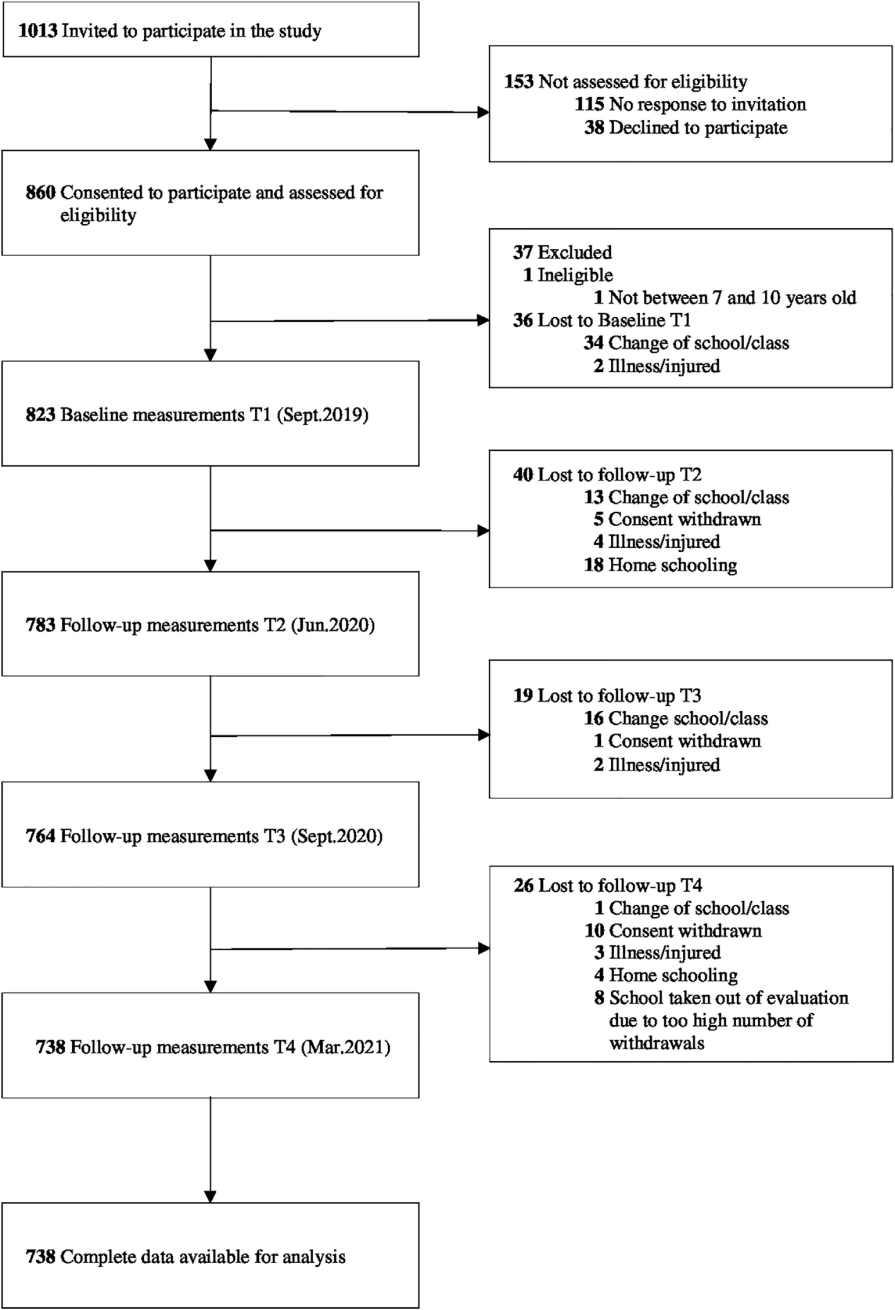
Flow diagram

By random number generator, 12 out of 39 primary schools from the districts of Klagenfurt (Austria) were selected. Following inclusion criteria were defined: age between 7 and 10 years, agreement to participate, and written consent of legal guardians. Out of 1013 invited school children, parental consent was obtained from 860 (84.9%) (Figure 1).

### 2.2 Procedure

In September 2019, baseline measures (T1) were carried out and were followed by further test phases in June 2020 (T2), September 2020 (T3), and March 2021 (T4). These follow‐up investigations were performed in strict compliance with the national guidelines of COVID‐19 measures. A detailed description of the restrictions relevant for primary school‐age children and the Oxford COVID‐19 Government Response Tracker, which provides internationally comparable stringency levels for Austria during the study period, is shown in the Data S1 (Tables S1 and S2, and Figure S1).

### 2.3 Outcomes

The primary outcome parameter of this study is the change of standardized BMI and associated weight classifications. Standing height was measured to the nearest 0.1 cm with a mobile stadiometer (SECA 213, Hamburg, Germany). Weight was determined in kilogram using an electronic scale (BOSCH PPW4202/01, Nuremberg, Germany).

Standardized values for BMI were calculated based on recent national^16^ and international (IOTF)^17^ age‐ and sex‐specific references. Using the Austrian national reference data, absolute BMI values were transferred into EQUI BMI_AUT_ values as described previously,^16^, ^18^ thus allowing direct comparison at different ages. Briefly, EQUI BMI_AUT_ curves allow projecting actual BMI into the cut‐offs at age 18 years relevant to define the risk for cardiovascular complications in later life (overweight: BMI >25 kg/m^2^, obesity BMI > 30 kg/m^2^, and morbid obesity BMI > 35 kg/m^2^). The same method of EQUI BMI_AUT_ was applied to define the categories normal weight (BMI 18.5–25 kg/m^2^) and underweight (16–18.5 kg/m^2^). Additionally, standard deviation scores (SDS) were calculated for international references as well (BMI_IOTF_ SDS). Standard deviation scores for height were calculated based on recent national reference values derived from the same sample as the BMI reference values.^19^

Alternative international reference values (WHO)^20^ were used in sensitivity analyses for BMI and height to check the robustness of the findings (Methods in the Supplements).

Secondary analyses were performed for possible influences of the COVID‐19 restrictions on height SDS, and for subgroups according to school location (urban vs. rural) and sex. The definition of Eurostat urban‐rural typology^21^ was used, based on the population density (inhabitants per km^2^). Urban areas were defined as those with more than 300 inhabitants per km^2^, whereas rural areas lie below this cut‐off. The demographics of the community in which the school was located were used to classify urban and rural schools.^22^, ^23^

### 2.4 Statistical analysis

For descriptive statistics, continuous variables are expressed as means (*M*) and standard deviations (SD), and categorical variables as absolute values (*n*) and percentages (%). No data imputation was performed.

#### 2.4.1 Changes over time

The changes in EQUI BMI_AUT_, BMI_IOTF_ SDS, and height SDS were analysed over the observation period by means of mixed‐design analyses of variance (ANOVAs) for sex, school location, and the time points September 2019, June 2020, September 2020, and March 2021. Harley's *F*‐max test was used to test for homogeneity. The Greenhouse–Geisser adjustment was used to correct sphericity violations. For ANOVAs, partial eta squared (*η*_*p*_^2^) was used to determine the size of the effect (≥0.01 = small, ≥0.06 = medium, and ≥0.14 = large),^24^ and only small effect was considered relevant.

Changes over time in the distribution of the BMI classification (five groups) were tested with the Friedman test. Post‐hoc tests were performed with the Wilcoxon signed‐rank test. All tests were two‐tailed, with a *p*‐value <0.05 considered statistically significant. Bonferroni correction was used for post‐hoc tests. All statistical calculations were performed using SPSS Version 27 (IBM Corp).

## 3 RESULTS

In September 2019, 823 children participated in baseline measurements. Eighty‐five children did not complete all four measurement time points and were excluded from the analyses, resulting in 738 children with complete anthropometric data (Figure 1). The included study population and the loss‐at‐follow‐up group were compared for the variables of age, sex, school location, BMI, and height. Children who were lost to follow‐up were less often from urban schools, but no other differences were found (Table S3). Sample characteristics are reported in the Data S1 (Table S4). In the total sample, the mean age at baseline was 8.3 ± 0.7 years (range: 7–10 years), 368 (49.9%) were girls, and 447 (60.6%) children visited schools in an urban location.

### 3.1 Change in BMI

BMI, weight classification, and height for the total sample are reported in Table 1. Between September 2019 and March 2021, EQUI BMI_AUT_ and BMI_IOTF_ SDS levels increased significantly, showing an increase of EQUI BMI_AUT_ from 22.28 to 22.92 (main effect time: *η*_*p*_^2^ = 0.080; *p* < 0.001) and an increase of BMI_IOTF_ SDS from 0.38 to 0.62 (main effect time: *η*_*p*_^2^ = 0.101; *p* < 0.001). Boys (EQUI BMI_AUT_ + 0.96 (95% CI, 0.80–1.13); BMI_IOTF_ SDS +0.35 (95% CI, 0.30–0.41)) showed a significantly larger increase in BMI than girls (EQUI BMI_AUT_ + 0.31 (95% CI, 0.19–0.43); BMI_IOTF_ SDS +0.14 (95% CI, 0.10–0.17)) with a small interaction effect over time (EQUI BMI_AUT_: time*sex: *η*_*p*_^2^ = 0.024; p < 0.001; BMI_IOTF_ SDS: time*sex: *η*_*p*_^2^ = 0.024; *p* < 0.001) (Tables 1, 2, and S5). Using the national reference values, children from urban schools (EQUI BMI_AUT_ + 0.82 [95% CI, 0.68–0.96]) showed a significantly higher increase in BMI than those from rural schools (EQUI BMI_AUT_ + 0.35 [95% CI, 0.20–0.50]) with a small interaction effect over time (EQUI BMI_AUT_: time*school location: *η*_*p*_^2^ = 0.012; p < 0.001) (Tables 1, 2, and S5). No differences were found between urban and rural schools using the IOTF reference values (Table 2).

**TABLE 1:** BMI and weight classification of the total sample, and by school location and gender

	Sep. 19	June 2020	Sep.20	Mar. 21
EQUI BMI_AUT_, Mean (SD)				
All	22.28 (3.55)	22.59 (3.79)	22.70 (3.86)	22.92 (3.99)
Urban schools	22.35 (3.68)	22.72 (4.09)	22.86 (4.13)	23.18 (4.31)
Rural school	22.18 (3.34)	22.40 (3.27)	22.48 (3.41)	22.52 (3.41)
Girls	22.15 (3.49)	22.31 (3.60)	22.39 (3.70)	22.45 (3.75)
Boys	22.42 (3.61)	22.88 (3.96)	23.02 (4.00)	23.38 (4.17)
AUT weight classification, No. (%)				
Underweight	44 (6.0)	37 (5.0)	32 (4.3)	32 (4.3)
Normal weight	582 (78.9)	571 (77.4)	565 (76.6)	555 (75.2)
Overweight	79 (10.7)	93 (12.6)	98 (13.3)	104 (14.1)
Obesity	26 (3.5)	28 (3.8)	32 (4.3)	33 (4.5)
Morbid obesity	7 (0.9)	9 (1.2)	11 (1.5)	14 (1.9)
BMI_IOTF_ SDS, Mean (SD)				
All	0.38 (1.09)	0.50 (1.11)	0.54 (1.10)	0.62 (1.08)
Urban schools	0.38 (1.11)	0.51 (1.17)	0.56 (1.14)	0.67 (1.11)
Rural school	0.37 (1.06)	0.49 (1.02)	0.51 (1.05)	0.54 (1.04)
Girls	0.47 (1.08)	0.55 (1.10)	0.57 (1.13)	0.61 (1.12)
Boys	0.28 (1.08)	0.45 (1.11)	0.51 (1.08)	0.64 (1.04)
IOTF weight classification, No. (%)				
Underweight	56 (7.6)	45 (6.1)	41 (5.6)	38 (5.1)
Normal weight	529 (71.7)	526 (71.3)	516 (69.9)	506 (68.6)
Overweight	99 (13.4)	103 (14.0)	117 (15.9)	124 (16.8)
Obesity	42 (5.7)	50 (6.8)	47 (6.4)	52 (7.0)
Morbid obesity	12 (1.6)	14 (1.9)	17 (2.3)	18 (2.4)
Height_AUT_ SDS, Mean (SD)				
All	0.27 (0.99)	0.31 (1.00)	0.31 (0.99)	0.33 (1.00)
Urban schools	0.24 (1.02)	0.24 (1.03)	0.27 (1.02)	0.28 (1.02)
Rural school	0.32 (0.95)	0.41 (0.96)	0.37 (0.96)	0.42 (.97)
Girls	0.32 (1.03)	0.37 (1.05)	0.38 (1.03)	0.43 (1.04)
Boys	0.22 (0.96)	0.25 (0.96)	0.23 (0.96)	0.24 (0.96)

*Note*: Study population, *N* = 738, data are no (%) or mean (SD). weight classification = underweight = equivalent BMI ≤ 18.5, normal weight = equivalent BMI = 18.5 to 25, overweight = equivalent BMI = 25.0 to 30.0, obesity = equivalent BMI = 30 to 35, morbid obesity = equivalent BMI > 35.

Abbreviations: BMI, body mass index; BMI_IOTF_ SDS, BMI SDS based on International Obesity Taskforce reference centile curves^18^; EQUI BMI_AUT_, equivalent BMI based on Austrian reference centile curves passing through adult BMI values^16^; Height_AUT_ SDS, height SDS based on Austrian reference centile curves^19^; SD, standard deviation; SDS, standard deviation score.

**TABLE 2:** 3‐way mixed ANOVAs for BMI and height SDS using Austrian and IOTF reference values

		Effects	df	F	*p* value	*η*_*p*_^2^	Power^a^
EQUI BMI_AUT_	Between‐subjects effects	Sex	1	4.00	0.046	0.005	0.52
		School location	1	1.53	0.22	0.002	0.24
		Sex*school location	1	0.10	0.75	<0.001	0.06
		Error	734				
	Within‐subjects effects	Time (T1‐T2‐T3‐T4)	2.55	64.10	<0.001	0.080	>0.99
		Time*sex	2.55	18.37	<0.001	0.024	>0.99
		Time*school location	2.55	8.95	<0.001	0.012	0.99
		Time*sex*school location	2.55	0.28	0.91	<0.001	0.10
		Error (Time)	1871.72				
Height_AUT_ SDS	Between‐subjects effects	Sex	1	3.90	0.049	0.005	0.51
		School location	1	2.37	0.12	0.003	0.34
		Sex*school location	1	1.31	0.25	0.002	0.21
		Error	734				
	Within‐subjects effects	Time (T1‐T2‐T3‐T4)	2.32	39.33	<0.001	0.051	>0.99
		Time*sex	2.32	16.53	<0.001	0.022	>0.99
		Time*school location	2.32	18.63	<0.001	0.025	>0.99
		Time*sex*school location	2.32	1.30	0.28	0.002	0.30
		Error (time)	1701.29				
BMI_IOTF_ SDS	Between‐subjects effects	Sex	1	1.21	0.27	0.002	0.20
		School location	1	0.51	0.48	0.001	0.11
		Sex*school location	1	0.03	0.87	<0.001	0.05
		Error	734				
	Within‐subjects effects	Time (T1‐T2‐T3‐T4)	2.74	82.28	<0.001	0.101	>0.99
		Time*sex	2.74	17.75	<0.001	0.024	>0.99
		Time*school location	2.74	5.54	0.001	0.007	0.93
		Time*sex*school location	2.74	0.59	0.61	0.001	0.17
		Error (time)	2013.32				

Abbreviations: *η*_*p*_^2^, partial eta square; ANOVA, analysis of variance; BMI, body mass index; BMI_IOTF_ SDS, BMI SDS based on International Obesity Taskforce reference centile curves^18^; df, degrees of freedom; EQUI BMI_AUT_, equivalent BMI based on Austrian reference centile curves passing through adult BMI values^16^; Height_AUT_ SDS, height SDS based on Austrian reference centile curves^19^; SDS, standard deviation score.

^a^ Observed power computed using alpha = 0.05.

### 3.2 Change in weight classification

**FIGURE 2:**
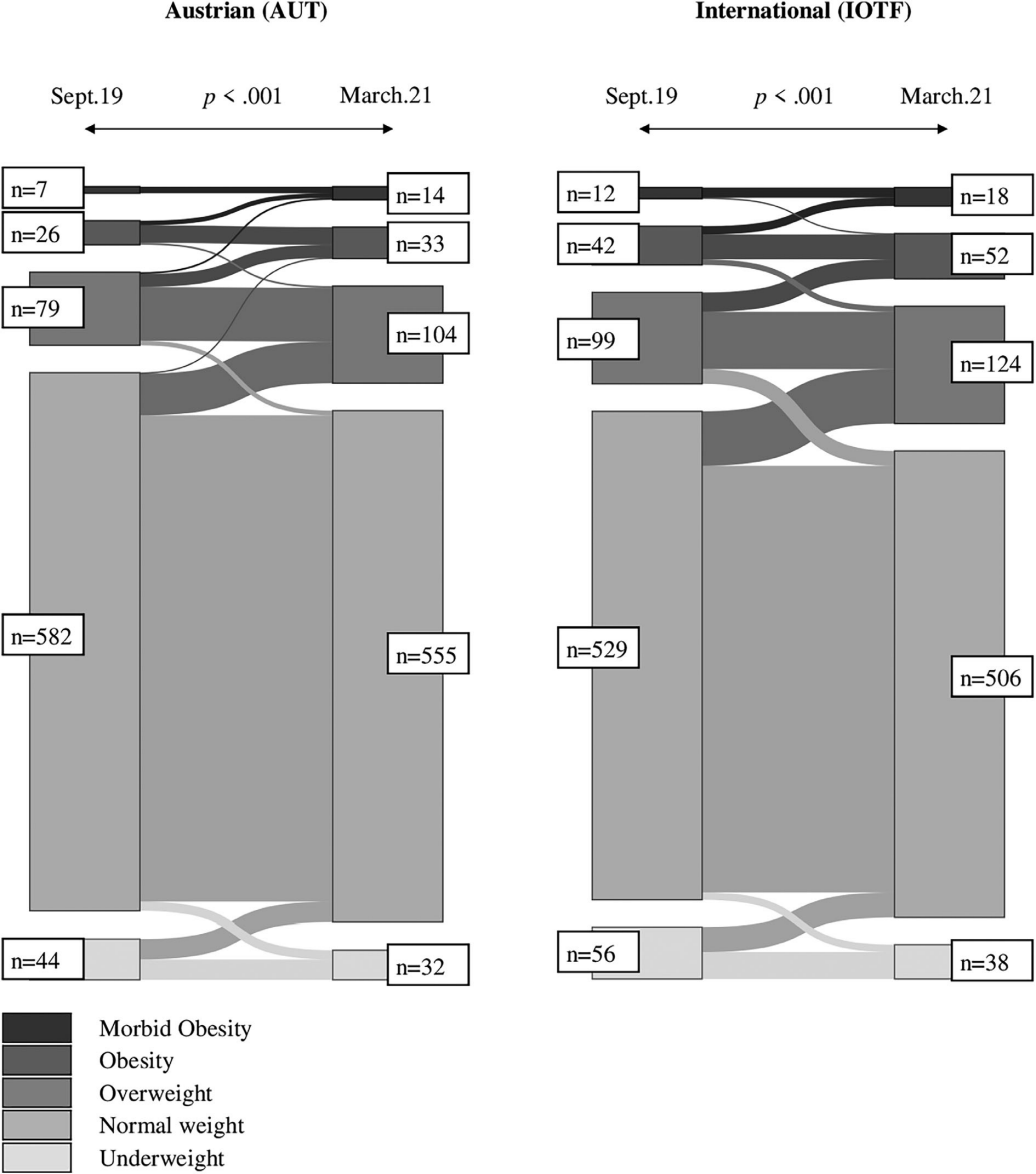
Changes in weight classification using a Sankey diagram (based on national and international reference values)

**FIGURE 3:**
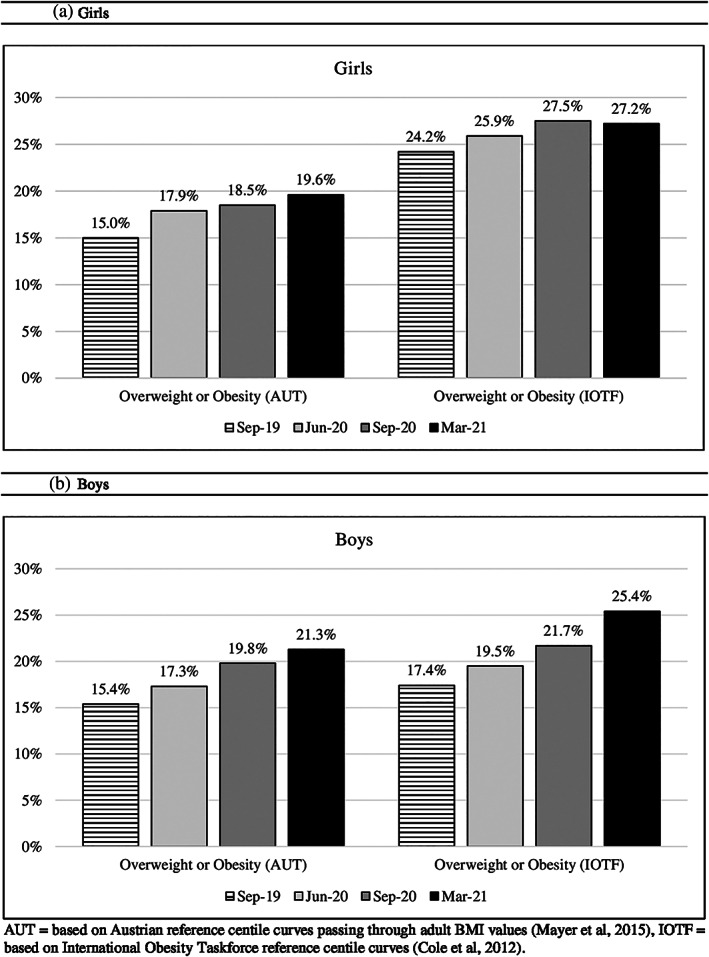
Percentage of children with overweight or obesity for national (AUT) and international (IOTF) cut‐off points at different time points

During the observation period September 2019 (T1) to March 2021 (T4), the number of children with overweight, obesity, or morbid obesity increased significantly over time (AUT: 15.1% to 20.5%, *p* < 0.001; IOTF: 20.7% to 26.2%, *p* < 0.001) (Figure 2 and Table 1). This increase was more pronounced in boys (AUT: +5.9% (15.4% to 21.3%), *p* < 0.001; IOTF: +8.0% (17.4% to 25.4%), *p* < 0.001) than in girls (AUT: +4.6% (15.0 to 19.6%), *p* = 0.020; IOTF: +3.0% (24.2% to 27.2%), *p* = 0.08) (Figure 3A,B and Tables S4, S6 & S7). The number of children with underweight or normal weight decreased (Figure 2 and Tables 1, S6, & S7).

### 3.3 Change in height

**FIGURE 4:**
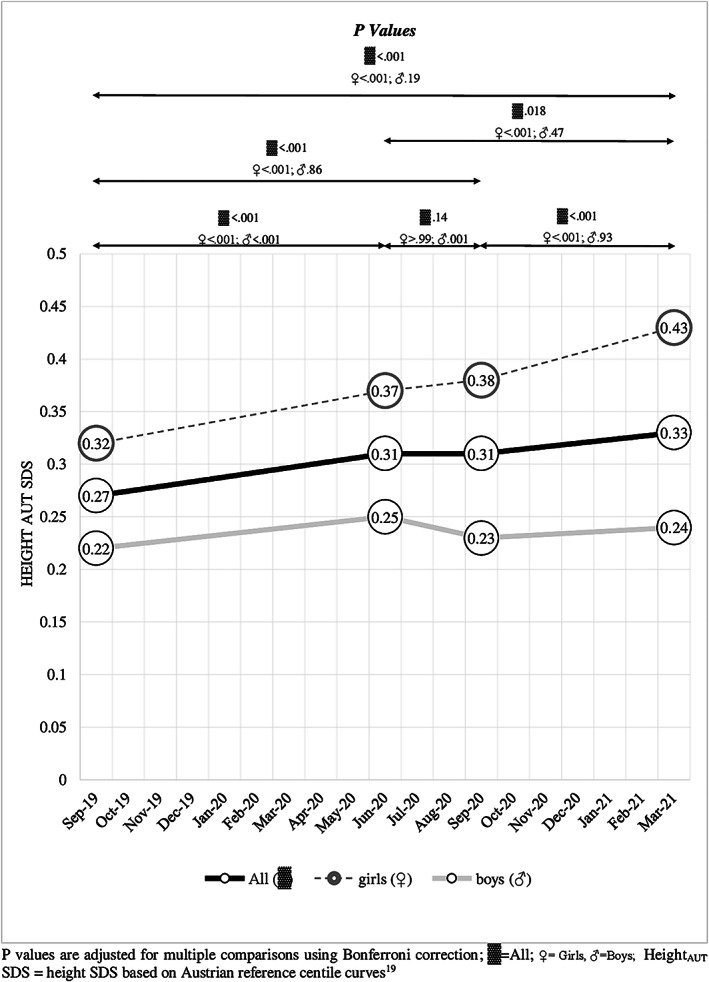
Increase of height SDS over time based on national reference values

During the observation period, a significant increase in height SDS from 0.27 to 0.33 (+0.06; 95% CI, 0.05–0.08) was observed in the total group (main effect time: *η*_*p*_^2^ = 0.051; *p* < 0.001) (Figure 4, Tables 1, 2, and S5). Interaction effects between time and sex (*η*_*p*_^2^ = 0.022; *p* < 0.001) and time and school location (*η*_*p*_^2^ = 0.025; *p* < 0.001) were found. Girls (+0.11, *p* < 0.001; 95% CI, 0.08–0.14) and children in rural schools (+0.09; *p* < 0.001; 95% CI, 0.06–0.13) showed a larger increase in height SDS than boys (+0.03; *p* = 0.19; 95% CI, −0.01 to 0.06) and children in urban schools (+0.04; *p* < 0.001; 95% CI, 0.02–0.07), respectively (Figure 4 and Tables 2 and S5).

Comparable trends for BMI SDS and height SDS were found when using WHO reference values (Tables S8, S9 & S10).

## 4 DISCUSSION

The findings of this cohort study demonstrate an increase in standardized mean BMI scores in 7‐ to 10‐year‐old children and consequently, a worrying increase in the proportion of children with overweight or obesity during the COVID‐19 pandemic.

Our results are in line with recent studies from various countries reporting an accelerated increase in BMI SDS during the COVID‐19 pandemic.^25^, ^26^, ^27^, ^28^, ^29^ The large increase in the number of children with overweight or obesity between September 2019 and March 2021 in our study is most likely associated with (repeated) closures of schools and sporting facilities. Whereas the causal pathways are complex and difficult to assess, drastic changes in weight‐related behaviour, like physical activity, sleep, screen exposure, and diet, have been observed in children during lockdowns.^8^, ^30^ The here reported increase in BMI is likely the combined effect of reduced physical activity,^31^, ^32^ increased sedentary behaviour,^7^, ^33^, ^34^ and changes in diet.^8^, ^35^, ^36^

Extending beyond the weight‐related findings, our data point to possible additional associations with growth. The significant increase in height SDS in girls was most pronounced during both lockdowns. The larger increase in height SDS in girls might also explain their smaller increase in BMI SDS compared to boys. Why girls increased in height SDS is difficult to explain. In our sample, changes in height SDS and BMI over time were not or only weakly correlated (data not shown). Nevertheless, a link between obesity and growth/tall stature has for a long time been discussed and reviewed in the literature. Putative effects of obesity on growth include involvement of growth factors (e.g., Insulin‐like growth factor‐I, insulin) and the role of an earlier onset of puberty. Both estrogens and androgens via aromatization to estrogens lead to the initiation of the pubertal and “pre‐pubertal” (adrenarche) growth spurt. This effect of body composition on human sexual development was already observed at the start of the “obesity pandemic.” However, a higher incidence of true precocious puberty was also reported during the COVID‐19 pandemic.^37^ We speculate that the significant increase in height SDS in girls could be a marker of the earlier activity of adrenal or gonadal sex steroids on the growth plate. Since pubertal development has not been assessed in our study population, this hypothesis has to be investigated in future studies including long‐term follow‐up of puberty, growth, and bone age as well as metabolic parameters/risk profiles.

### 4.1 Strengths and limitations

Our study is representative of primary school children in the region of Klagenfurt (Austria) and considers rural and urban areas in equal measure. There was a very high participation rate and a low dropout rate so that the available results can be generalized for regions where similar COVID‐19 restrictions have been imposed. All data were collected objectively in healthy children beginning prior to the pandemic and are not self‐reported.

As a limitation, our study was originally designed as an intervention program, and COVID‐19 changed the objective. Due to the general COVID‐19 restrictions on the whole population, recruiting a control group was impossible. The results can therefore only be interpreted with regard to established sex‐ and age‐specific national and international reference standards..^16^, ^17^, ^19^, ^20^

### 4.2 Implications

John F. Kennedy said, “There is only one thing that is more expensive in the long run than education, no education.” At the time of this statement, an obesity epidemic was still a long way off, and it can therefore be assumed that President Kennedy was referring primarily to intellectual education with subject‐related knowledge transfer and not physical education. Our study shows the changes in BMI in primary school‐aged children when relevant physical priming/programming for further life takes place. The increase in BMI may have negative effects on chronic disease risk later in life and, therefore, might affect general health in the population and our health system in the future.

It is necessary to avoid future closures of schools and sporting facilities to prevent further negative effects on the physical health of children. Above that, it will be necessary to create versatile sport and physical activity opportunities, and to support children in improving their eating behaviour, to counteract these negative side effects of COVID‐19 mitigation measures. In the educational setting, support measures have already been implemented to reduce the negative effects in the main subjects (mathematics, language), but such support measures for supporting children to improve their lifestyle behaviours are lacking. Such support measures should be taken as quickly as possible.

## 5 CONCLUSION

In a representative sample of primary school children in Austria, BMI and the proportion of children with overweight or obesity increased significantly during the concurrent COVID‐19 pandemic. A larger increase was observed in boys than in girls. The situation after 1 year of the implementation of the COVID‐19 restrictions is worrying and long‐term adverse consequences are likely. To counteract, targeted intervention programs focusing on physical activity and a healthy diet should be implemented immediately. The simultaneously observed increase in growth in girls is remarkable and warrants further follow‐up of their height and weight development.

## CONFLICT OF INTEREST

No conflict of interest was declared.

## AUTHOR CONTRIBUTIONS

All authors had full access to all the data in the study and take responsibility for the integrity of the data and the accuracy of the data analysis. *Concept and design*: Gerald Jarnig and Mireille N.M. van Poppel; *Acquisition, analysis, or interpretation of data*: Gerald Jarnig, Johannes Jaunig, Reinhold Kerbl, Gabriele Häusler, Volker Strenger and Mireille N.M. van Poppel. *Drafting of the manuscript*: Gerald Jarnig, Johannes Jaunig and Mireille N.M. van Poppel. *Critical revision of the manuscript for important intellectual content*: Gerald Jarnig, Johannes Jaunig, Reinhold Kerbl, Gabriele Häusler, Volker Strenger and Mireille N.M. van Poppel. *Statistical analysis*: Gerald Jarnig, Johannes Jaunig and Mireille N.M. van Poppel. *Obtained funding*: Gerald Jarnig. *Administrative, technical, or material support*: Gerald Jarnig. *Supervision*: Gerald Jarnig, Johannes Jaunig, Reinhold Kerbl, Gabriele Häusler, Volker Strenger, Mireille N.M. van Poppel.

## Supporting information


**Data S1.** Supporting Information.
