# Supplementary material for: Acceleration in BMI gain following COVID‐19 restrictions. A longitudinal study with 7‐ to 10‐year‐old primary school children
Source: Pediatr Obes. 2022 Jan 26;17(6):e12890. doi: 10.1111/ijpo.12890 (PMC9286467; doi:10.1111/ijpo.12890)
Supplement: Supplementary file 1 — Data S1. Supporting Information. [file IJPO-17-0-s001.pdf]

Acceleration in BMI gain following COVID-19 restrictions. A longitudinal study with 7- to 10-year-old primary school children.

Supplementary material

Gerald Jarnig[1], Johannes Jaunig[1], Reinhold Kerbl[2], Volker Strenger[3], Gabriele Häusler[4], Mireille N.M. van Poppel[1]

Affiliations:

[1]Institute of Human Movement Science, Sport and Health, University of Graz, Graz, Austria

[2]Department of Pediatrics and Adolescent Medicine, LKH Hochsteiermark/Leoben, Austria

[3] Department of Pediatrics and Adolescent Medicine, Medical University of Graz, Austria

[4]Department of Pediatrics and Adolescent Medicine, Division of Pulmonology, Allergology and Endocrinology- Vienna Bone and Growth Center, Medical University of Vienna, Austria

Corresponding Author:

Gerald Jarnig

Institute of Human Movement Science, Sport and Health,

University of Graz

Mozartgasse 14

8010 Graz, Austria

gerald.jarnig@gmx.at

+43 (0) 316 380 8326

This supplementary material has been provided by the authors to give readers additional information about their work.

Additional methods

Table S1. Restriction levels for children in Austria from January 31, 2020 to March 31, 2021 in relation to the OxCGRT stringency index

Table S2. Detailed description of the restrictions for Austrian children in primary school from January 31, 2020 to March 31, 2021 in relation to the OxCGRT stringency index

Table S3. Overall sample characteristics study population vs. loss at follow-up

Table S4. Additional sample characteristics for boys and girls and school location

Table S5. Post-hoc tests for BMI and Height SDS for the main effect time and interactions for time*sex and time*school location based on the estimated marginal means for Austrian and IOTF reference values

Table S6. Friedman test for weight classification using Austrian and IOTF cut offs to baseline measurements T1 and follow-up measurements T2, T3 and T4

Table S7. Post-hoc analyses by the Wilcoxon test for weight classifications using Austrian and IOTF cut offs

Table S8. BMI SDS, weight classification and height SDS according to WHO reference values

Table S9. 3-way mixed ANOVAs for BMI SDS and height SDS using WHO reference values

Table S10. Post-hoc tests for BMI SDS and height SDS for the main effect time and interactions for time*sex and time*school location based on the estimated marginal means for WHO reference values

Figure S1. COVID-19 restrictions in Austria between January 31, 2020 and March 31, 2021

Figure S1A. Restriction levels for primary school children.

Figure S1B. OxCGRT - Stringency Index.

Additional methods

To check for robustness of the main outcomes, sensitivity analyses were conducted. Alternative international references values were used for body mass index (BMI) and height.

Standardization of the body mass index and height

For comparison with alternative international reference values, the WHO[1] references were considered, whereas the calculations were based on the LMS method.[2] The WHO reference values are expressed in SDS (referred to as BMI_{IOTF} SDS and $Height_{WHO}$ SDS).

Table S1. Restriction levels for children in Austria from January 31, 2020 to March 31, 2021 in relation to the OxCGRT stringency index

Period	OxCGRT Austria	School structure	Physical education in school	Sports and leisure facilities	Sport at the club	Stay at home require ments	Sum of restrictions in the school
01-31-2020 – 02-23-2020	0,00	0	0	0	0	0	0
02-24-2020 – 03-08-2020	11,11	0	0	0	0	0	0
03-09-2020 – 03-11-2020	19,44	0	0	0	0	0	0
03-12-2020	34,26	0	0	0	0	0	0
03-13-2020 – 03-15-2020	48,15	0	0	0	0	0	0
03-16-2020 – 04-13-2020	81,48	3	3	3	3	3	15
04-14-2020 – 04-22-2020	77,78	3	3	3	3	3	15
04-23-2020 – 04-30-2020	75,00	3	3	3	3	3	15
05-01-2020	67,59	3	3	2	3	2	13
05-02-2020 – 05-10-2020	64,81	3	3	2	3	2	13
05-11-2020 – 05-17-2020	59,26	3	3	2	3	2	13
05-18-2020 – 05-28-2020	59,26	2	3	2	3	2	12
05-29-2020 – 06-02-2020	53,70	2	3	2	2	2	11
06-03-2020	50,00	2	3	2	2	2	11
06-04-2020 – 07-01-2020	47,22	2	3	2	2	2	11
07-02-2020 – 07-09-2020	47,22	2	3	0	1	0	6
07-10-2020 – 09-05-2020	35,19	0	0	0	1	0	1
09-06-2020 – 09-13-2020	36,11	0	0	0	1	0	1
09-14-2020 – 09-16-2020	36,11	1	0	0	1	1	3
09-17-2020 – 09-28-2020	37,04	1	0	0	1	1	3
09-29-2020 – 10-12-2020	40,74	1	0	0	1	1	3
10-13-2020 – 10-16-2020	44,91	1	0	0	1	2	4
10-17-2020 – 10-22-2020	58,80	1	0	0	1	2	4
10-23-2020	60,19	1	0	0	1	2	4
10-24-2020 – 10-26-2020	60,19	0	0	1	3	2	6
10-27-2020 – 11-01-2020	64,81	0	0	1	3	2	6
11-02-2020	75,00	0	0	1	3	2	6
11-03-2020 – 11-16-2020	75,00	0	2	1	3	2	8
11-17-2020 – 12-06-2020	82,41	3	3	1	3	2	12
12-07-2020 – 12-23-2020	71,30	1	2	1	3	1	8
12-24-2020 – 12-25-2020	82,41	3	3	1	3	2	12
12-26-2020 – 01-06-2021	82,41	3	3	1	3	2	12
01-07-2021 – 01-18-2021	82,41	3	3	1	3	2	12
01-19-2021 – 02-05-2021	82,41	2	2	1	3	1	9
02-06-2021 – 02-07-2021	82,41	3	3	1	3	1	11
02-08-2021 – 02-11-2021	74,07	3	3	1	3	1	11
02-12-2021 – 02-14-2021	79,63	3	3	1	3	1	11
02-15-2021 – 03-14-2021	75,93	1	2	1	3	1	8
03-15-2021 – 03-31-2021	73,15	1	2	1	2	1	7

Level 0 = no restrictions, Level 1 = low restrictions, Level 2 = medium restrictions, Level 3 = high restrictions, OxCGRT = Oxford COVID-19 Government Response Tracker Stringency Index

Table S2. Detailed description of the restrictions for Austrian children in primary school from January 31, 2020 to March 31, 2021 in relation to the OxCGRT stringency index

Period	OxCGRT Austria	School structure	Physical education in school	Sports and leisure facilities	Sport at the club	Stay at home requirements
01-31-2020 – 02-23-2020	0,00	A	A	A	A	A
02-24-2020 – 03-08-2020	11,11	A	A	A	A	A
03-09-2020 – 03-11-2020	19,44	A	A	A	A	A
03-12-2020	34,26	A	A	A	A	A
03-13-2020 – 03-15-2020	48,15	A	A	A	A	A
03-16-2020 – 04-13-2020	81,48	B	D	E	G	I
04-14-2020 – 04-22-2020	77,78	B	D	E	G	I
04-23-2020 – 04-30-2020	75,00	B	D	E	G	I
05-01-2020	67,59	B	D	F	G	J
05-02-2020 – 05-10-2020	64,81	B	D	F	G	J
05-11-2020 – 05-17-2020	59,26	B	D	F	G	J
05-18-2020 – 05-28-2020	59,26	C	D	F	G	J
05-29-2020 – 06-02-2020	53,70	C	D	F	H	J
06-03-2020	50,00	C	D	F	H	J
06-04-2020 – 07-01-2020	47,22	C	D	F	H	J
07-02-2020 – 07-09-2020	47,22	C	D	A	L	A
07-10-2020 – 09-05-2020	35,19	K	K	A	L	A
09-06-2020 – 09-13-2020	36,11	K	K	A	L	A
09-14-2020 – 09-16-2020	36,11	M	A	A	L	J
09-17-2020 – 09-28-2020	37,04	M	A	A	L	J
09-29-2020 – 10-12-2020	40,74	M	A	A	L	J
10-13-2020 – 10-16-2020	44,91	M	A	A	L	O
10-17-2020 – 10-22-2020	58,80	M	A	A	L	O
10-23-2020	60,19	M	A	A	L	O
10-24-2020 – 10-26-2020	60,19	K	K	F	G	O
10-27-2020 – 11-01-2020	64,81	K	K	F	G	O
11-02-2020	75,00	K	K	F	G	O
11-03-2020 – 11-16-2020	75,00	M	N	F	G	O
11-17-2020 – 12-06-2020	82,41	B	D	F	G	O
12-07-2020 – 12-23-2020	71,30	M	N	F	G	J
12-24-2020 – 12-25-2020	82,41	K	K	F	G	O
12-26-2020 – 01-06-2021	82,41	K	K	F	G	O
01-07-2021 – 01-18-2021	82,41	B	D	F	G	O
01-19-2021 – 02-05-2021	82,41	C	N	F	G	J
02-06-2021 – 02-07-2021	82,41	K	K	F	G	J
02-08-2021 – 02-11-2021	74,07	K	K	F	G	J
02-12-2021 – 02-14-2021	79,63	K	K	F	G	J
02-15-2021 – 03-14-2021	75,93	M	N	F	G	J
03-15-2021 – 03-31-2021	73,15	M	N	F	H	J

OxCGRT = Oxford COVID-19 Government Response Tracker Stringency Index , A = no restrictions, B = Distance learning in primary school, C = Lessons in primary school in shifts / home schooling are possible and recommended, D = no physical education in school, E = Closures of all public playgrounds and sports facilities, F = public playgrounds and sports grounds are reopened, minimum distance must be maintained, G = Club sport is forbidden, H = Club sport is possible under severe restrictions and conditions, I = Required with exceptions, J = Restricted freedom of movement / keeping a minimum distance; K = holidays, L = Practice of club sports is allowed under submission of prevention concepts, M = ordinary lessons in primary school / home schooling possible, N = Limited sports activities in elementary school, O = Recommended to stay at home.

Table S3. Overall sample characteristics study population vs. loss at follow-up

	**Study population**	**loss at follow-up**	X^2	**t**	***P* value**
**Age (years)**	8.3 (0.7)	8.1 (0.7)		1.725	.09
**Female Sex**	368 (49.1%)	51 (60.0%)	3.133		.07
**Urban region**	447 (60.6%)	38 (44.7%)	7.925		.005
**EQUI BMI_{AUT}**	22.29 (3.55)	22.73 (3.27)		1.385	.17
**$Height_{AUT}$ SDS**	0.27 (0.99)	0.13 (1.11)		1.173	.24
**BMI_{IOTF} SDS**	0.38 (1.09)	0.21 (1.07)		1.378	.17

Sample size for study population, N = 738; for loss at follow up, N = 85.

Data are n (%) or mean (SD). EQUI BMI_{AUT} = equivalent BMI based on Austrian reference centile curves passing through adult BMI values (Mayer et al, 2015), $Height_{AUT}$ SDS = height SDS based on Austrian reference centile curves (Gleiss et al, 2013), BMI_{IOTF} SDS = BMI SDS based on International Obesity Taskforce reference centile curves (Cole et al, 2012), X^2 = Chi-Square Test value; t = test statistic t-test; BMI = body mass index.

Table S4. Additional sample characteristics for boys and girls and school location

			Sep-19	June 2020	Sep-20	Mar-21
**Age (years), Mean (SD)**			8.3 (0.7)	9.0 (0.7)	9.2 (0.7)	9.7 (0.7)
**Female Sex, No. (%)**			368 (49.9%)			
**Urban school, No. (%)**			447 (60.6%)			
**Girls, No. (%)**	urban schools		212 (57.6%)			
	rural schools		156 (42.4%)			
**Boys, No. (%)**	urban schools		235 (63.5%)			
	rural schools		135 (36.5%)			
**AUT weight classification, No. (%)**	**Girls**	Underweight	22 (6.0)	22 (6.0)	24 (6.5)	26 (7.1)
		Normal weight	291 (79.1)	280 (76.1)	276 (75.0)	270 (73.4)
		Overweight	40 (10.9)	53 (14.4)	50 (13.6)	54 (14.7)
		Obesity	12 (3.3)	9 (2.4)	14 (3.8)	14 (3.8)
		Morbid obesity	3 (0.8)	4 (1.1)	4 (1.1)	4 (1.1)
	**Boys**	Underweight	22 (5.9)	15 (4.1)	8 (2.2)	6 (1.6)
		Normal weight	291 (78.6)	291 (78.6)	289 (78.1)	285 (77.0)
		Overweight	39 (10.5)	40 (10.8)	48 (13.0)	50 (13.5)
		Obesity	14 (3.8)	19 (5.1)	18 (4.9)	19 (5.1)
		Morbid obesity	4 (1.1)	5 (1.4)	7 (1.9)	10 (2.7)
	**Urban schools**	Underweight	27 (6.0)	25 (5.6)	19 (4.3)	19 (4.3)
		Normal weight	350 (78.3)	337 (75.4)	337 (75.4)	325 (72.7)
		Overweight	48 (10.7)	57 (12.8)	58 (13.0)	71 (15.9)
		Obesity	17 (3.8)	21 (4.7)	25 (5.6)	21 (4.7)
		Morbid obesity	5 (1.1)	7 (1.6)	8 (1.8)	11 (2.5)
	**Rural schools**	Underweight	17 (5.8)	12 (4.1)	13 (4.5)	13 (4.5)
		Normal weight	232 (79.7)	234 (80.4)	228 (78.4)	230 (790)
		Overweight	31 (10.7)	36 (12.4)	40 (13.7)	33 (11.3)
		Obesity	9 (3.1)	7 (2.4)	7 (2.4)	12 (4.1)
		Morbid obesity	2 (0.7)	2 (0.7)	3 (1.0)	3 (1.0)
**IOTF weight classification, No. (%)**	**Girls**	Underweight	24 (6.5)	23 (6.3)	23 (6.3)	27 (7.3)
		Normal weight	255 (69.3)	250 (67.9)	244 (66.3)	241 (65.5)
		Overweight	58 (15.8)	61 (16.6)	69 (18.8)	64 (17.4)
		Obesity	24 (6.5)	26 (7.1)	22 (6.0)	26 (7.1)
		Morbid obesity	7 (1.9)	8 (2.2)	10 (2.7)	10 (2.7)
	**Boys**	Underweight	32 (8.6)	22 (5.9)	18 (4.9)	11 (3.0)
		Normal weight	274 (74.1)	276 (74.6)	272 (73.5)	265 (71.6)
		Overweight	41 (11.1)	42 (11.4)	48 (13.0)	60 (16.2)
		Obesity	18 (4.9)	24 (6.5)	25 (6.8)	26 (7.0)
		Morbid obesity	5 (1.4)	6 (1.6)	7 (1.9)	8 (2.2)
	**Urban schools**	Underweight	34 (7.6)	32 (7.2)	23 (5.1)	21 (4.7)
		Normal weight	319 (71.4)	307 (68.7)	307 (68.7)	299 (66.9)
		Overweight	57 (12.8)	65 (14.5)	73 (16.3)	81 (18.1)
		Obesity	28 (6.3)	32 (7.2)	31 (6.9)	32 (7.2)
		Morbid obesity	9 (2.0)	11 (2.5)	13 (2.9)	14 (3.1)
	**Rural schools**	Underweight	22 (7.6)	13 (4.5)	18 (6.2)	17 (5.8)
		Normal weight	210 (72.2)	219 (75.3)	209 (71.8)	207 (71.1)
		Overweight	42 (14.4)	38 (13.1)	44 (15.1)	44 (15.1)
		Obesity	14 (4.8)	18 (6.2)	16 (5.5)	19 (6.5)
		Morbid obesity	3 (1.0)	3 (1.0)	4 (1.4)	4 (1.4)

Study population, N = 738, Data are No (%) or Mean (SD). AUT weight classification = based on Austrian reference centile curves passing through adult BMI values (Mayer et al, 2015), IOTF weight classification = based on International Obesity Taskforce reference centile curves (Cole et al, 2012); Weight classification = Underweight = equivalent BMI = < 18.5, Normal weight = equivalent BMI = 18,5 to 25, Overweight = equivalent BMI = 25.0 to 30.0, Obesity = equivalent BMI = 30 to 35, Morbid obesity = equivalent BMI > 35; (Mayer et al, 2015; Cole et al, 2012).

Table S5. Post-hoc tests for BMI and Height SDS for the main effect time and interactions for time*sex and time*school location based on the estimated marginal means for Austrian and IOTF reference values

		**Pairwise comparisons**	**Mean diff (95% CI)**	**SE**	**p-lvl**	***P* Value[a]**
**EQUI BMI$_{AUT}$**	Time	T1 vs T2	-0.296 (-0.413 to -0.178)	0.044	***	<.001
		T1 vs T3	-0.404 (-0.530 to -0.278)	0.048	***	<.001
		T1 vs T4	-0.590 (-0.729 to -0.452)	0.052	***	<.001
		T2 vs T3	-0.109 (-0.209 to -0.009)	0.038	*	.024
		T2 vs T4	-0.295 (-0.408 to -0.182)	0.043	***	<.001
		T3 vs T4	-0.186 (-0.277 to -0.095)	0.034	***	<.001
	Time*Sex (Girls)	T1 vs T2	-0.147 (-0.311 to 0.018)	0.062		.11
		T1 vs T3	-0.229 (-0.405 to -0.052)	0.067	**	.004
		T1 vs T4	-0.267 (-0.462 to -0.073)	0.073	**	.002
		T2 vs T3	-0.082 (-0.221 to 0.058)	0.053		.73
		T2 vs T4	-0.121 (-0.278 to 0.037)	0.060		.26
		T3 vs T4	-0.039 (-0.166 to 0.089)	0.048		>.99
	Time*Sex (Boys)	T1 vs T2	-0.444 (-0.613 to -0.276)	0.064	***	<.001
		T1 vs T3	-0.580 (-0.761 to -0.400)	0.068	***	<.001
		T1 vs T4	-0.913 (-1.112 to -0.715)	0.075	***	<.001
		T2 vs T3	-0.136 (-0.279 to 0.007)	0.054		.07
		T2 vs T4	-0.469 (-0.631 to -0.308)	0.061	***	<.001
		T3 vs T4	-0.333 (-0.464 to -0.203)	0.050	***	<.001
	Time*School Location (Urban)	T1 vs T2	-0.353 (-0.501 to -0.206)	0.056	***	<.001
		T1 vs T3	-0.495 (-0.653 to -0.336)	0.060	***	<.001
		T1 vs T4	-0.809 (-0.983 to -0.635)	0.066	***	<.001
		T2 vs T3	-0.141 (-0.267 to -0.016)	0.047	*	.018
		T2 vs T4	-0.455 (-0.597 to -0.314)	0.053	***	<.001
		T3 vs T4	-0.314 (-0.429 to -0.200)	0.043	***	<.001
	Time*School Location (Rural)	T1 vs T2	-0.238 (-0.421 to -0.054)	0.069	**	.004
		T1 vs T3	-0.314 (-0.511 to -0.118)	0.074	***	<.001
		T1 vs T4	-0.372 (-0.588 to -0.156)	0.082	***	<.001
		T2 vs T3	-0.077 (-0.232 to 0.079)	0.059		>.99
		T2 vs T4	-0.135 (-0.310 to 0.041)	0.066		.26
		T3 vs T4	-0.058 (-0.200 to 0.084)	0.054		>.99
**Height$_{AUT}$ SDS**	Time	T1 vs T2	-0.050 (-0.066 to -0.033)	0.006	***	<.001
		T1 vs T3	-0.038 (-0.057 to -0.020)	0.007	***	<.001
		T1 vs T4	-0.069 (-0.091 to -0.046)	0.008	***	<.001
		T2 vs T3	0.012 (-0.002 to 0.025)	0.005		.14
		T2 vs T4	-0.019 (-0.035 to -0.002)	0.006	*	.018
		T3 vs T4	-0.030 (-0.045 to -0.016)	0.005	***	<.001
	Time*Sex (Girls)	T1 vs T2	-0.058 (-0.081 to -0.035)	0.009	***	<.001
		T1 vs T3	-0.062 (-0.088 to -0.035)	0.010	***	<.001
		T1 vs T4	-0.111 (-0.143 to -0.080)	0.012	***	<.001
		T2 vs T3	-0.004 (-0.023 to 0.015)	0.007		>.99
		T2 vs T4	-0.053 (-0.077 to -0.030)	0.009	***	<.001
		T3 vs T4	-0.050 (-0.070 to -0.029)	0.008	***	<.001
	Time*Sex (Boys)	T1 vs T2	-0.042 (-0.066 to -0.018)	0.009	***	<.001
		T1 vs T3	-0.015 (-0.042 to 0.012)	0.010		.86
		T1 vs T4	-0.026 (-0.058 to 0.006)	0.012		.19
		T2 vs T3	0.027 (0.008 to 0.046)	0.007	**	.001
		T2 vs T4	0.016 (-0.008 to 0.040)	0.009		.47
		T3 vs T4	-0.011 (-0032 to 0.010)	0.008		.93
	Time*School Location (Urban)	T1 vs T2	-0.006 (-0.026 to 0.015)	0.008		>.99
		T1 vs T3	-0.032 (-0.055 to -0.008)	0.009	**	.003
		T1 vs T4	-0.043 (-0.082 to -0.004)	0.015	*	.021
		T2 vs T3	-0.026 (-0.043 to -0.009)	0.006	***	<.001
		T2 vs T4	-0.038 (-0.072 to -0.003)	0.013	*	.024

		T3 vs T4	-0.012 (-0.044 to 0.021)	0.012		>.99
	Time*School Location (Rural)	T1 vs T2	-0.094 (-0.120 to -0.069)	0.010	***	<.001
		T1 vs T3	-0.045 (-0.075 to -0.016)	0.011	***	<.001
		T1 vs T4	-0.094 (-0.129 to -0.059)	0.013	***	<.001
		T2 vs T3	0.049 (0.028 to 0.070)	0.008	***	<.001
		T2 vs T4	-0.000 (-0.026 to 0.026)	0.010		>.99
		T3 vs T4	-0.049 (-0.072 to -0.027)	0.008	***	<.001
**BMI$_{IOTF}$ SDS**	Time	T1 vs T2	-0.126 (-0.169 to -0.082)	0.016	***	<.001
		T1 vs T3	-0.161 (-0.206 to -0.117)	0.017	***	<.001
		T1 vs T4	-0.236 (-0.279 to -0.192)	0.017	***	<.001
		T2 vs T3	-0.036 (-0.075 to 0.003)	0.015		.09
		T2 vs T4	-0.110 (-0.150 to -0.070)	0.015	***	<.001
		T3 vs T4	-0.074 (-0.106 to -0.042)	0.012	***	<.001
	Time*Sex (Girls)	T1 vs T2	-0.081 (-0.142 to -0.021)	0.023	**	.003
		T1 vs T3	-0.100 (-0.162 to -0.038)	0.023	***	<.001
		T1 vs T4	-0.125 (-0.186 to -0.064)	0.023	***	<.001
		T2 vs T3	-0.019 (-0.073 to 0.036)	0.021		>.99
		T2 vs T4	-0.044 (-0.099 to 0.012)	0.021		.23
		T3 vs T4	-0.025 (-0.069 to 0.019)	0.017		.821
	Time*Sex (Boys)	T1 vs T2	-0.170 (-0.232 to -0.107)	0.024	***	<.001
		T1 vs T3	-0.223 (-0.286 to -0.160)	0.024	***	<.001
		T1 vs T4	-0.346 (-0.409 to -0.284)	0.024	***	<.001
		T2 vs T3	-0.053 (-0.109 to 0.003)	0.021		.07
		T2 vs T4	-0.176 (-0.233 to -0.119)	0.022	***	<.001
		T3 vs T4	-0.123 (-0.169 to -0.078)	0.017	***	<.001

a adjusted for multiple comparisons using Bonferroni correction.
p-lvl (P Value level) * = P <.05, ** = P <.01, *** = P <.001, BMI = body mass index, CI = confidence interval, EQUI BMI$_{AUT}$ = equivalent BMI based on Austrian reference centile curves passing through adult BMI values (Mayer et al, 2015), Height$_{AUT}$ SDS= height SDS based on Austrian reference centile curves (Gleiss et al, 2013), BMI$_{IOTF}$ SDS = BMI SDS based on International Obesity Taskforce reference centile curves (Cole et al, 2012), Mean diff = mean difference based on the estimated marginal means, p-lvl = significance level, SDS = standard deviation score, SE = standard error, T1= baseline measurements in Sept and Oct 2019, T2 = follow-up measurements in May and Jun 2020, T3 = follow-up measurements in Sep and Oct 2020, T4 = follow-up measurements in Feb and Mar 2021.

Table S6. Friedman test for weight classification using Austrian and IOTF cut offs to baseline measurements T1 and follow-up measurements T2, T3 and T4

	**n**	**df**	**X^2**	**p-lvl**	***P* Value**
**AUT weight classification**	738	3	68.945	***	<.001
**AUT weight classification, Girls**	368	3	9.886	*	.020
**AUT weight classification, Boys**	370	3	69.488	***	<.001
**IOTF weight classification**	738	3	64.350	***	<.001
**IOTF weight classification, Girls**	368	3	6.659		.08
**IOTF weight classification, Boys**	370	3	79.976	***	<.001

n = Study population, df = degrees of freedom, X2 = Chi-Square Test value,
p-lvl (P Value level) * = P <.05, ** = P <.01, *** = P <.001;
AUT weight classification = based on Austrian reference centile curves passing through adult BMI values (Mayer et al, 2015), IOTF weight classification = based on International Obesity Taskforce reference centile curves (Cole et al, 2012); T1= baseline measurements in Sept and Oct 2019, T2 = follow-up measurements in May and Jun 2020, T3 = follow-up measurements in Sep and Oct 2020, T4 = follow-up measurements in Feb and Mar 2021.

Table S7. Post-hoc analyses by the Wilcoxon test for weight classifications using Austrian and IOTF cut offs

	Pairwise comparisons	Z[a]	p-lvl	*P* Value[b]
**AUT weight classification, All**	T2 to T1	-3.444	**	.003
	T3 to T1	-5.897	***	<.001
	T4 to T1	-6.742	***	<.001
	T3 to T2	-2.869	*	.025
	T4 to T2	-4.500	***	<.001
	T4 to T3	-2.109		.21
**AUT weight classification, Girls**	T2 to T1	-1.768		.46
	T3 to T1	-2.785	*	.032
	T4 to T1	-2.592		.06
	T3 to T2	-.845		>.99
	T4 to T2	-1.183		>.99
	T4 to T3	-.333		>.99
**AUT weight classification, Boys**	T2 to T1	-3.000	*	.016
	T3 to T1	-5.252	***	<.001
	T4 to T1	-6.521	***	<.001
	T3 to T2	-3.212	**	.008
	T4 to T2	-4.907	***	<.001
	T4 to T3	-2.785	*	.032
**IOTF weight classification, All**	T2 to T1	-3.450	**	.003
	T3 to T1	-5.167	***	<.001
	T4 to T1	-6.727	***	<.001
	T3 to T2	-2.251		.15
	T4 to T2	-4.315	***	<.001
	T4 to T3	-2.495		.08
**IOTF weight classification, Girls**	T2 to T1	-1.364		>.99
	T3 to T1	-2.109		.21
	T4 to T1	-1.969		.29
	T3 to T2	-.926		>.99
	T4 to T2	-.687		>.99
	T4 to T3	-.164[c]		>.99
**IOTF weight classification, Boys**	T2 to T1	-3.677	**	.001
	T3 to T1	-5.250	***	<.001
	T4 to T1	-7.313	***	<.001
	T3 to T2	-2.236		.15
	T4 to T2	-5.461	***	<.001
	T4 to T3	-3.464	**	.003

a = Based on negative ranks, b = adjusted for multiple comparisons using Bonferroni correction, c = Based on positive ranks. Study population, N = 738; Z = Test Statistic; p-lvl (P Value level) * = P <.05, ** = P <.01, *** = P <.001; AUT weight classification = based on Austrian reference centile curves passing through adult BMI values (Mayer et al, 2015), IOTF weight classification = based on International Obesity Taskforce reference centile curves (Cole et al, 2012); T1= baseline measurements in Sept and Oct 2019, T2 = follow-up measurements in May and Jun 2020, T3 = follow-up measurements in Sep and Oct 2020, T4 = follow-up measurements in Feb and Mar 2021.

Table S8. BMI SDS, weight classification and height SDS according to WHO reference values

			**Sep.19**	**June 2020**	**Sep.20**	**Mar-21**
**BMI$_{WHO}$ SDS, Mean (SD)**	All		0.36 (1.22)	0.50 (1.25)	0.55 (1.25)	0.64 (1.23)
	urban schools		0.37 (1.25)	0.51 (1.32)	0.57 (1.29)	0.70 (1.26)
	rural school		0.34 (1.18)	0.48 (1.14)	0.51 (1.18)	0.55 (1.17)
**Height$_{WHO}$ SDS, Mean (SD)**	All		0.63 (0.96)	0.65 (0.97)	0.65 (0.96)	0.70 (0.98)
	urban schools		0.60 (0.99)	0.59 (1.00)	0.62 (0.99)	0.65 (1.00)
	rural school		0.67 (0.90)	0.75 (0.92)	0.70 (0.92)	0.78 (.94)
**WHO weight classification, No. (%)**	All	Underweight	7 (0.9)	11 (1.5)	7 (0.9)	3 (0.4)
		Normal weight	533 (72.2)	499 (67.6)	495 (67.1)	487 (66.0)
		Overweight	117 (15.9)	131 (17.8)	138 (18.7)	137 (18.6)
		Obesity	81 (11.0)	97 (13.1)	98 (13.3)	111 (15.0)
	Urban schools	Underweight	4 (0.9)	9 (2.0)	3 (0.7)	1 (0.2)
		Normal weight	319 (71.4)	297 (66.4)	296 (66.2)	290 (64.9)
		Overweight	72 (16.1)	78 (17.4)	84 (18.8)	80 (17.9)
		Obesity	52 (11.6)	63 (14.1)	64 (14.3)	76 (17.0)
	Rural schools	Underweight	3 (1.0)	2 (0.7)	4 (1.4)	2 (0.7)
		Normal weight	214 (73.5)	202 (69.4)	199 (68.4)	197 (67.7)
		Overweight	45 (15.5)	53 (18.2)	54 (18.6)	57 (19.6)
		Obesity	29 (10.0)	34 (11.7)	34 (11.7)	35 (12.0)

Study population, N = 738, Data are No. (%) or Mean (SD), BMI$_{WHO}$ SDS = Standard deviation score based on World Health Organization reference centile curves (de Onis et al, 2007), Height$_{WHO}$ SDS = Standard deviation score based on World Health Organization reference centile curves (de Onis et al, 2007), BMI = body mass index; Weight classification = Underweight = BMI$_{WHO}$ < -2.0, Normal weight = BMI$_{WHO}$ -2.0 to +1.0, Overweight = BMI$_{WHO}$ >1.0 to 2.0, Obesity = BMI$_{WHO}$ > 2.0, (WHO Multicentre Growth Reference Study Group, 2006);

Table S9. 3-way mixed ANOVAs for BMI SDS and height SDS using WHO reference values

		**Effects**	**df**	**F**	***P* Value**	**η_p^2**	**Power[a]**
**BMI_{WHO} SDS**	Between-subjects effects	Sex	1	0.001	.97	<.001	.05
		School Location	1	0.502	.48	.001	.11
		Sex*School Location	1	0.039	.84	<.001	.06
		Error	734				
	Within-subjects effects	Time (T1-T2-T3-T4)	2.73	85.400	<.001	.104	>.99
		Time*Sex	2.73	22.232	<.001	.029	>.99
		Time*School Location	2.73	4.933	.003	.007	.89
		Time*Sex*School Location	2.73	0.586	.57	.001	.17
		Error (Time)	2000.91				
**$Height_{WHO}$ SDS**	Between-subjects effects	Sex	1	0.88	.35	.001	.16
		School Location	1	2.29	.13	.003	.33
		Sex*School Location	1	1.35	.25	.002	.21
		Error	734				
	Within-subjects effects	Time (T1-T2-T3-T4)	2.22	61.13	<.001	.077	>.99
		Time*Sex	2.22	5.97	.002	.008	.90
		Time*School Location	2.22	23.27	<.001	.031	>.99
		Time*Sex*School Location	2.22	1.00	.37	.001	.24
		Error (Time)	1626.35				

[a] observed power computed using alpha = .05

ANOVA = analysis of variance, BMI = body mass index, SDS = Standard Deviation Score, df = degrees of freedom, η_p^2 = partial eta square. BMI_{WHO} SDS = Standard deviation score based on World Health Organization reference centile curves (de Onis et al, 2007), $Height_{WHO}$ SDS = Standard deviation score based on World Health Organization reference centile curves (de Onis et al, 2007).

Table S10. Post-hoc tests for BMI SDS and height SDS for the main effect time and interactions for time*sex and time*school location based on the estimated marginal means for WHO reference values

		**Pairwise comparisons**	**Mean diff (95% CI)**	**SE**	**p-lvl**	***P* Value[a]**
**BMI$_{WHO}$ SDS**	Time	T1 vs T2	-0.142 (-0.191 to -0.094)	0.018	***	<.001
		T1 vs T3	-0.185 (-0.235 to -0.135)	0.019	***	<.001
		T1 vs T4	-0.269 (-0.319 to -0.220)	0.019	***	<.001
		T2 vs T3	-0.042 (-0.087 to 0.001)	0.016		.06
		T2 vs T4	-0.127 (-0.171 to -0.083)	0.017	***	<.001
		T3 vs T4	-0.084 (-0.120 to -0.048)	0.013	***	<.001
	Time*Sex (Girls)	T1 vs T2	-0.081 (-0.149 to -0.013)	0.026	**	.010
		T1 vs T3	-0.104 (-0.174 to -0.034)	0.026	***	<.001
		T1 vs T4	-0.130 (-0.199 to -0.061)	0.026	***	<.001
		T2 vs T3	-0.023 (-0.084 to 0.038)	0.023		>.99
		T2 vs T4	-0.049 (-0.111 to -0.013)	0.023		.23
		T3 vs T4	-0.026 (-0.076 to 0.024)	0.019		>.99
	Time*Sex (Boys)	T1 vs T2	-0.204 (-0.273 to -0.134)	0.026	***	<.001
		T1 vs T3	-0.266 (-0.338 to -0.195)	0.027	***	<.001
		T1 vs T4	-0.408 (-0.479 to -0.338)	0.027	***	<.001
		T2 vs T3	-0.063 (-0.125 to 0.000)	0.024	*	.047
		T2 vs T4	-0.142 (-0.268 to -0.142)	0.024	***	<.001
		T3 vs T4	-0.142 (-0.193 to -0.091)	0.019	***	<.001
**Height$_{WHO}$ SDS**	Time	T1 vs T2	-0.032 (-0.047 to -0.016)	0.006	***	<.001
		T1 vs T3	-0.020 (-0.038 to -0.003)	0.007	*	.014
		T1 vs T4	-0.080 (-0.102 to -0.059)	0.008	***	<.001
		T2 vs T3	0.011 (-0.001 to 0.0023)	0.005		.08
		T2 vs T4	-0.049 (-0.065 to -0.033)	0.006	***	<.001
		T3 vs T4	-0.060 (-0.074 to -0.047)	0.005	***	<.001
	Time*School Location (Urban)	T1 vs T2	0.014 (-0.005 to 0.033)	0.007		.33
		T1 vs T3	-0.015 (-0.037 to 0.007)	0.008		.39
		T1 vs T4	-0.053 (-0.092 to -0.013)	0.015	**	.002
		T2 vs T3	-0.029 (-0.044 to -0.014)	0.006	***	<.001
		T2 vs T4	-0.067 (-0.102 to -0.032)	0.013	***	<.001
		T3 vs T4	-0.037 (-0.071 to -0.004)	0.013	*	.018
	Time*School Location (Rural)	T1 vs T2	-0.077 (-0.101 to -0.053)	0.009	***	<.001
		T1 vs T3	-0.025 (-0.052 to -0.002)	0.010		.09
		T1 vs T4	-0.108 (-0.142 to -0.074)	0.013	***	<.001
		T2 vs T3	0.052 (0.033 to 0.071)	0.007	***	<.001
		T2 vs T4	-0.031 (-0.057 to -0.006)	0.010	**	.007
		T3 vs T4	-0.083 (-0.104 to -0.062)	0.008	***	<.001

a adjusted for multiple comparisons using Bonferroni correction.

p-lvl (P Value level) * = P <.05, ** = P <.01, *** = P <.001, BMI = body mass index, CI = confidence interval, BMI$_{WHO}$ SDS and Height$_{WHO}$ SDS = Standard deviation score (SDS) based on World Health Organization reference centile curves (de Onis et al, 2007), Mean diff = mean difference based on the estimated marginal means, p-lvl = significance level, SDS = standard deviation score, SE = standard error, T1= baseline measurements in Sept and Oct 2019, T2 = follow-up measurements in May and Jun 2020, T3 = follow-up measurements in Sep and Oct 2020, T4 = follow-up measurements in Feb and Mar 2021.

Figure S1. COVID-19 restrictions in Austria between January 31, 2020 and March 31, 2021

Figure S1A. Restriction levels for primary school children.

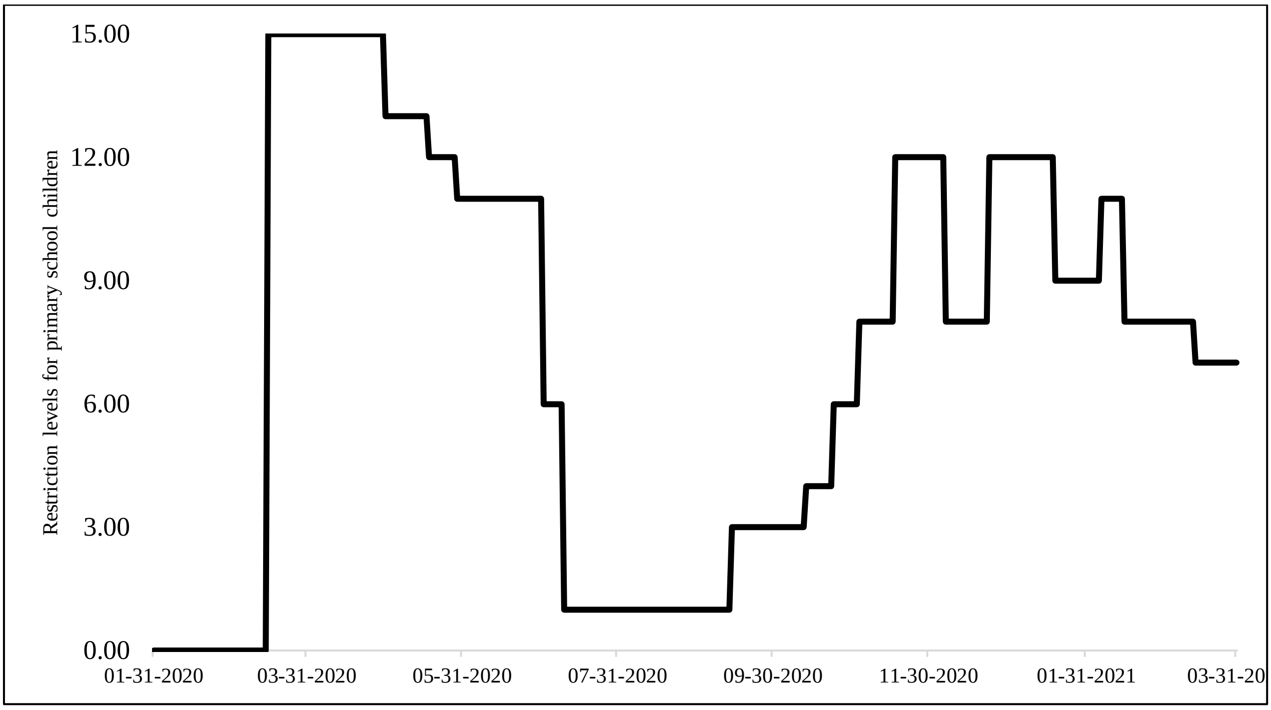

Figure S1B. OxCGRT - Stringency Index.

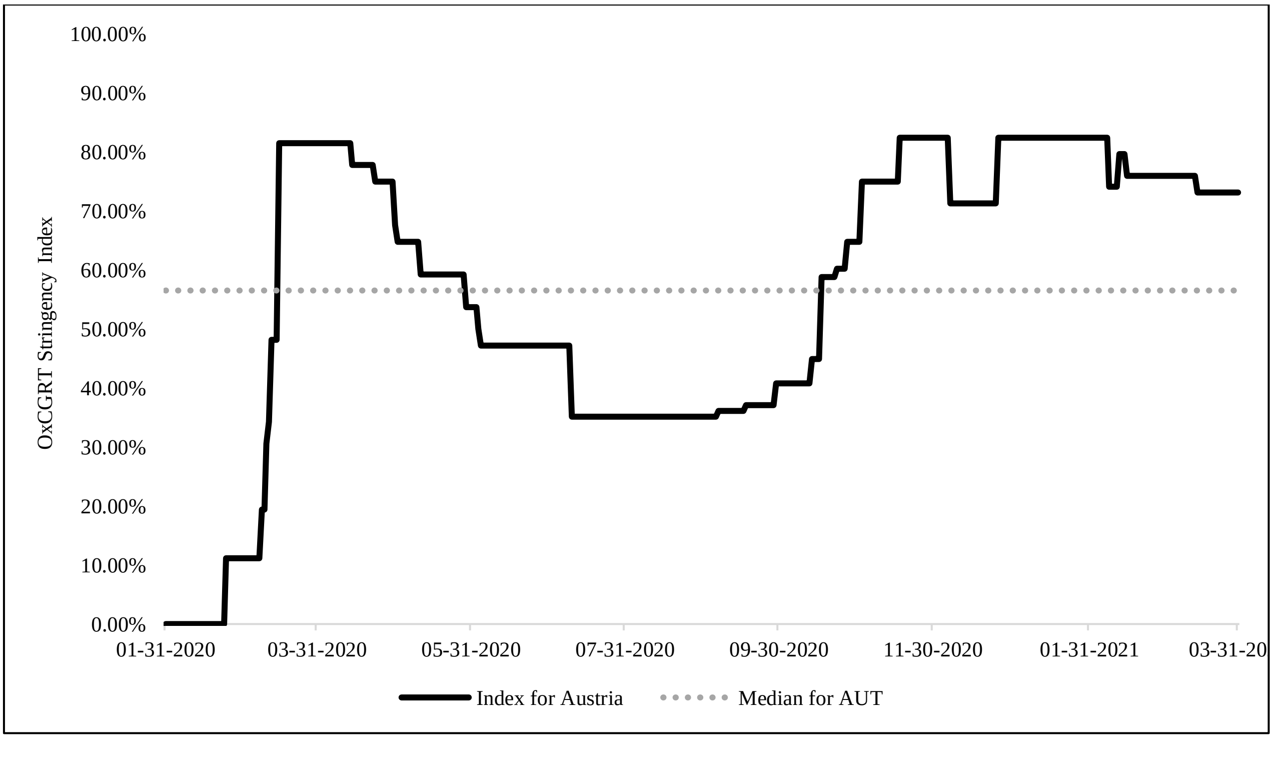

The detailed description of this classification method used in Figure S1A is available in Table S1 and Table S2. Figure S1B shows the Oxford COVID-19 Government Response Tracker (OxCGRT) Stringency Index for Austria between January 31, 2020 and March 31, 2021.

References

1. de Onis M, Onyango AW, Borghi E, Siyam A, Nishida C, Siekmann J. Development of a WHO growth reference for school-aged children and adolescents. *Bull World Health Organ*. 2007;85(9):660-667. doi:10.2471/blt.07.043497
2. Cole TJ, Bellizzi MC, Flegal KM, Dietz WH. Establishing a standard definition for child overweight and obesity worldwide: international survey. *BMJ*. 2000;320(7244):1240-1243. doi:10.1136/bmj.320.7244.1240
